# Potent cytotoxic effects of *Calomeria amaranthoides *on ovarian cancers

**DOI:** 10.1186/1756-9966-30-29

**Published:** 2011-03-14

**Authors:** Caroline van Haaften, Colin C Duke, Arij M Weerheim, Nico PM Smit, Paul MM van Haard, Firouz Darroudi, Baptist JMZ Trimbos

**Affiliations:** 1 Department of Gynaecology, Leiden University Medical Center, The Netherlands; 2 Faculty of Pharmacy, University of Sydney, NSW 2006, Australia; 3 Skin Research Laboratory, Leiden University Medical Center, Leiden, The Netherlands; 4 Department of Clinical Chemistry, Leiden University Medical Center, Leiden, The Netherlands; 5 Department of Clinical Chemistry, Medical Laboratories, Reinier de Graaf Group of Hospitals, Delft, The Netherlands; 6 Department of Toxicogenetics, Leiden University, Medical Center Leiden, The Netherlands

## Abstract

**Background:**

Ovarian cancer remains the leading cause of death from gynaecological malignancy. More than 60% of the patients are presenting the disease in stage III or IV. In spite of combination of chemotherapy and surgery the prognosis stays poor for therapy regimen.

**Methods:**

The leaves of a plant endemic to Australia, *Calomeria amaranthoides*, were extracted and then fractionated by column chromatography. *In vitro *cytotoxicity tests were performed with fractions of the plant extract and later with an isolated compound on ovarian cancer cell lines, as well as normal fibroblasts at concentrations of 1-100 μg/mL (crude extract) and 1-10 μg/mL (compound). Cytotoxicity was measured after 24, 48 and 72 hours by using a non-fluorescent substrate, Alamar blue.

*In vivo *cytotoxicity was tested on ascites, developed in the abdomen of nude mice after inoculation with human OVCAR_3 _cells intraperitoneally. The rate of change in abdomen size for the mice was determined by linear regression and statistically evaluated for significance by the unpaired t test.

**Results:**

Two compounds were isolated by chromatographic fractionation and identified by ^1^H-NMR, ^13^C-NMR and mass spectrometry analyses, EPD, an α-methylene sesquiterpene lactone of the eremophilanolide subtype, and EPA, an α-methylene carboxylic acid.

Cytotoxicity of EPD for normal fibroblasts at all time points IC_50 _was greater than 10 μg/mL, whereas, for OVCAR_3 _cells at 48 hours IC_50 _was 5.3 μg/mL (95% confidence interval 4.3 to 6.5 μg/mL).

Both, the crude plant extract as well as EPD killed the cancer cells at a final concentration of 10 μg/mL and 5 μg/mL respectively, while in normal cells only 20% cell killing effect was observed. EPA had no cytotoxic effects.

Changes in abdomen size for control versus Cisplatin treated mice were significantly different, P = 0.023, as were control versus EPD treated mice, P = 0.025, whereas, EPD versus Cisplatin treated mice were not significantly different, P = 0.13.

**Conclusions:**

For the first time both crude plant extract from *Calomeria amaranthoides *and EPD have been shown to have potent anti-cancer effects against ovarian cancer.

## Background

*Calomeria amaranthoides*, described both by Ventenat and Smith in 1804 [1,2] as *Humea elegans *belonging to the genus *Haeckeria *in the tribe of Inuleae was grown in France and England from seeds originating from the Blue Mountains, New South Wales (NSW) in Australia. The plant is of a monotypic genus, endemic to NSW and Victoria, Australia [3].

In 2004 the genus *Haeckeria *was reassessed by Orchard as *C. amaranthoides *and since then *C. amaranthoides *belongs to the genus *Calomeria *of the family Asteraceae (Compositae) [4]. As a biennial plant it can grow to more than three metres high, with flowers as waving plume bushes and wrinkly leaves with an aromatic scent. It is also called incense plant.

The plant family of Asteraceae are known for their natural products. One type includes sesquiterpene lactones (SL) which to date is of great interest for their potential as anti-cancer agents as reviewed by Heinrich et al. and Zhang et al. [5,6].

Ovarian cancer is the fifth leading cause of death in women and remains the leading cause of death from gynaecological malignancy in many countries, in spite of chemotherapy with Platinum derivates and/or Taxol after surgery. Of the malignant epithelial tumors (>90% of all ovarian cancers), the serous papillary variants form the largest subgroup [7,8]. Due to its dismal prognosis there is an urgent need for new treatment strategy for ovarian cancer.

For the first time we have studied *C. amaranthoides *for its possible anti-tumor activity. An SL (EPD) and a structurally related sesquiterpene (EPA) have been found, extracted and purified. Among them EPD has shown *in vitro *and *in vivo *(mice) high toxicity in ovarian cancers.

## Methods

A voucher specimen of *Calomeria amaranthoides*, collected near Old Bell's Line of Road, Mount Tomah NSW 2758, Australia, is held in the John Ray Herbarium, University of Sydney, Collection number: Silvester 110118-01.

Leaves of *C. amaranthoides*, gathered in the Blue Mountains (Mount Tomah, NSW, Australia) were air-dried while protected from sunlight.

### Fractionation of extracts by column chromatography

Dried plant material (350 g), cut in small pieces was soaked in chloroform (CHCl_3_) at room temperature. After 24-48 hours a crude extract of the leaves was removed and evaporated under reduced pressure (21.3 grams, 6.0%). The residue, re-dissolved in CHCl_3 _(30 mL) was applied to a column (21 cm × 5 cm i.d.) filled with Silicagel (Lichroprep Si 60, particle size 15-25 μm; Merck, Germany). Elution was carried out with a stepwise gradient consisting of hexane:dioxane, 98:2 (v/v 400 mL); hexane:chloroform:dioxane, 88:10:2 (v/v 600 mL); hexane:chloroform:dioxane:ethyl acetate:2-propanol, 80:10:2:6:1, (v/v 600 mL) and hexane:chloroform:acetone:methanol, 56:20:16:8, (v/v 400 mL). A total of 157 fractions (10 mL each) were collected and combined into groups based on HPLC analysis. The combined group of fractions showing the highest toxicity towards ovarian cancer cells was further fractionated by short column vacuum chromatography.

### High-performance liquid chromatography (HPLC)

HPLC analyses were carried out using the Akta purifier (Amersham Pharmacia Biotech, Sweden) with a HPLC-column (150 mm × 4.6 mm i.d. plus pre-column; Grace, The Netherlands), filled with HS Silica (particle size 3 μm), UV detection at 214 nm, 254 nm and 280 nm. Ten μL of the fractionated extract was injected, after dilution to 100 μL with eluent A: hexane (99.5 mL)-dioxane (0.5 mL). The first 10 minutes the column was eluted at a flow rate of 0.5 mL/min with eluent A, followed by 30 minutes with eluent B: hexane (85 mL)-diethyl ether (10 mL)-ethanol (5 mL).

### ^1^H-NMR and ^13^C-NMR analyses

^1^H-NMR and ^13^C-NMR spectroscopy was performed on those plant fractions with clear cytotoxicity effects. ^1^H-NMR, ^13^C-NMR and Correlation Spectroscopy (COSY) were performed using a Varian Gemini 300 MHz instrument (Palo Alto, CA, USA). The spectra were measured in parts per million (ppm) and were referenced to tetramethylsilane (TMS = 0 ppm).

Electrospray ionisation in positive and negative mode (ESI) mass spectrometry analyses were performed using a TSQ 7000 Liquid Chromatography Mass Spectrometer (LC-MS/MS; Thermo, San Jose, CA, USA), equipped with Xcalibur data acquisition and processing software. Short-Column Vacuum Chromatography (SCVC) was performed using a column packed with TLC-grade silica gel H60 (Merck, Darmstadt, Germany)) and applying a step-wise gradient of solvents with increasing polarity. Substances were detected by TLC performed on silica gel coated TLC plates (H60 F254, Merck, Germany) and by ^1^H-NMR spectroscopy. Structures of purified compounds were determined by mass spectrometry and ^1^H-NMR and ^13^C-NMR spectroscopy.

### Graphs and Statistics

Graphing and statistical evaluations were carried out with GraphPad Prism 5 for Windows.

### Cell lines and cell cultures

Cells used in the assays were five ovarian cell lines (JV, JG, JC, JoN, NF), which were earlier established [9,10], two cell lines OVCAR_3 _and SKOV_3 _from the American Type Culture Collection (ATCC) as well as epithelial cells from the ovary (serous papillary cystadenomas) [11] and human dermal fibroblasts primary cultures [12].

### *In vitro *cytotoxicity tests with different fractions of *C. amaranthoides*

*In vitro *cytotoxicity tests were performed using a non-fluorescent substrate, Alamar blue (BioSource Invitrogen, UK), as described by Pagé et al. [13]. Ovary cells (1 × 10^4 ^or 5 × 10^4^) were seeded in 24-wells plates (Costar, USA) and grown in RPMI-1640, supplemented with 6 mM L-glutamine, 10% fetal calf serum (FCS) (Gibco, Invitrogen, UK) and penicillin (100 units/mL) and streptomycin (100 μg/mL), while normal fibroblasts were grown in Dulbecco's modified Eagle medium (DMEM), also supplemented with L-glutamine and FCS. The cultures were maintained in a humidified atmosphere of 5% CO_2 _at 37°C.

Cell cultures, in triplicates, in exponential growth were treated with the different dried fractions of the plant extract, redissolved in dimethyl sulfoxide (DMSO) and added at final concentrations of 1, 10 and 100 μg/mL. The control cultures had 0.02% (1 μg/mL) 0.2% (10 μg/mL) and 2% (100 μg/mL) DMSO added to the medium. In 2 mL medium/well 10% Alamar blue was added and 100 μl of the supernatants of the 24-well plates after 24, 48 and 72 hrs incubations were pipetted into 96-well plates (Costar, USA). Cell viability was measured with a 96-well plate reader (Molecular Devices Ltd, UK). In a later stage, after identifying fractions with high cytotoxic effects, the final concentrations of extracts tested ranged from 1-10 μg/mL, with final concentrations of 0.02 up to 0.2% DMSO.

### *In vivo *pilot experiment

An *in vivo *pilot experiment was performed with 20 BALB/c nude mice (Charles River Laboratories, France). In order to mimic advanced ovarian cancer the mice were injected intraperitoneally (i.p.) with 10^7 ^OVCAR_3 _cells (ATCC) into the abdominal cavity to form ascites. Three groups of mice were examined: 6 control mice (no treatment), 6 mice treated with Cisplatin and 6 mice treated with EPD after ascites had formed. Cells of ascites of two mice were frozen and stored for future experiments. To study reduction of the swollen abdomen 5 mg/kg Platosin (Cisplatin, Pharma Chemie, The Netherlands) and the isolated compound EPD at a final concentration of 20 mg/kg were administered i.p.

## Results

### Fractionation of extracts by column chromatography

In total 157 fractions were sampled and, based on HPLC analyses, divided into four groups of combined fractions (fractions: 1-6, 60-70, 90-100 and 120-130) and then tested *in vitro *against ovarian cancer cell lines and normal cells. Group 2 (fractions: 60-70) showed the strongest cytotoxicity, killing all ovarian cancer cells at 10 μg/mL but not at 1 μg/mL. Other fractions did not show significant activities. This second group of fractions 60-70 (1.30 g, 0.37% yield from crude extract) was further fractionated by normal-phase short-column vacuum chromatography on silica gel H (column dimensions 18 mm × 65 mm i.d.), eluted with stepwise solvent gradients of hexane: dichloromethane, 1:1 v/v (100 mL and 50 mL); dichloromethane (2 × 50 mL); dichloromethane: ethyl acetate, 4:1 v/v (2 × 50 mL); dichloromethane: ethyl acetate, 1:1 v/v (2 × 50 mL); ethyl acetate (2 × 50 mL). From each fraction (12 in total) solvent was evaporated under reduced pressure and the residue was weighed.

Bioassays with ovarian cancer cells indicated fraction 4 (309 mg, 0.09% of the dried plant; out of the twelve fractions, see above) as the fraction with most of the cytotoxicity and its main chemical constituent was identified as EPD. A second main non-cytotoxic constituent, present mostly in Fractions 7 to 9 was identified as EPA (137 mg, 91% purity by NMR and MS analyses).

Again, fractionation was applied to fraction 4 (enriched in EPD) using normal-phase short-column vacuum chromatography (silica gel H; column dimensions 18 mm × 65 mm i.d.), eluting with stepwise solvent gradients of hexane:dichloromethane, 2:1 v/v (100 mL); hexane: dichloromethane, 1:1 v/v (2 × 50 mL); hexane:dichloromethane, 1:2 v/v (2 × 50 mL); dichloromethane (2 × 50 mL); dichloromethane: ethyl acetate 4:1 (2 × 50 mL); dichloromethane: ethyl acetate, 1:1 v/v (2 × 50 mL) to give the main chemical constituent, identified as an SL, EPD (93 mg, 90% purity by NMR and MS analyses) and containing lipids and waxes (10% by NMR analyses).

A small sample of freshly dried leaves (1.63 g) was extracted with dichloromethane (100 mL), filtered and the dichloromethane removed under reduced pressure leaving a dark green residue (62.6 mg, yield 3.9%). Quantitative ^1^H-NMR analysis of a CDCl_3 _solution showed EPD 44%, EPA 31% and a complex mixture of unidentified constituents 25%.

A small sample of dried leaves (10.31 g), that had been stored in the dark under ambient conditions for 3.5 years was extracted with CHCl_3 _(100 mL, 48 hours) filtered and the CHCl_3 _removed under reduced pressure leaving a dark green-brown residue (0.62 g, yield 6.0%). Quantitative ^1^H-NMR analysis of a CDCl_3 _solution showed that EPD and EPA were almost completely absent and a very complex mixture of unidentified constituents made up the bulk of the material.

### ^1^H-NMR and ^13^C-NMR analyses

#### Eremophila-1(10)-11(13)-dien-12,8β-olide (EPD)

(3aα,4aα,5α,9aα)-3a,4,4a,5,6,7,9,9a-octahydro-4a,5-dimethyl-3-methylenenaphtho[2,3-*b*]furan-2(3*H*)-2-one

C_15_H_20_O_2 _colourless liquid; ^1^H-NMR (CDCl_3_): δ0.92 (s, H-14), 0.93 (d, *J*_4,15 _= 6.8 Hz, H-15), 1.50 (m, H-3), 1.60 (m, H-4), 1.70 (m, H-6), 2.03 (m, H-2), 2.30 (m, H-9), 2.58 (dd, *J*_9,9' _= 12.6 Hz, *J*_8,9' _= 7.7 Hz, H-9'), 2.92 (m, H-7), 4.53 (dt, *J*_7,8 _= 9.6 Hz, *J*_8,9 _= 7.4 Hz, H-8), 5.48 (br t, *J*_1,2 _= 3.4 Hz, H-1), 5.59 (d, *J*_13,13' _= 2.2 Hz, H-13'), 6.23 (d, *J*_13,13' _= 2.2 Hz, H-13); ^13^C-NMR (CDCl_3_): δ16.08, 20.59, 25.03, 26.72, 34.69, 34.91, 36.63, 37.01, 38.73, 79.00, 121.82, 124.57, 138.32, 139.36, 170.65. Positive ion ESI-MS [M+Na]^+ ^255 (100), [M+H]^+ ^233 (65). Xanthanodien or EPD is an α-methylene SL [14].

#### Eremophila-1(10),11(13)-dien-12-oic acid (EPA)

C_15_H_22_O_2 _colourless liquid; ^1^H-NMR (CDCl_3_): δ0.85 (d, *J*_4,15 _= 6.4 Hz, H-15), 0.91 (s, H-14), 1.45 (m, H-6), 1.50 (m, H-4), 1.55 (m, H-3), 1.60 (m, H-8), 1.85 (m, H-9), 2.01 (m, H-2), 2.40 (m, H-9'), 2.55 (m, H-7), 5.38 (br t, *J*_1,2 _= 3.4 Hz, H-1), 5.66 (br s, H-13'), 6.29 (br s, H-13); ^13^C-NMR (CDCl_3_): δ16.08, 20.59, 25.03, 26.72, 34.69, 34.91, 36.63, 37.01, 38.73, 79.00, 121.82, 124.57, 138.32, 139.36, 170.65. Negative ion ESI-MS [M-H]^- ^233 (100)

EPA, is an α-methylene carboxylic acid [15].

**Figure 1:**
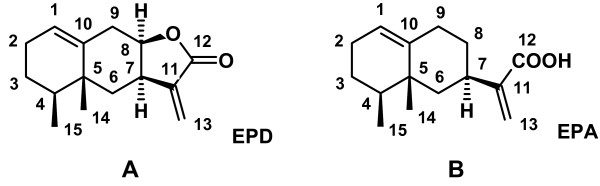
**Chemical structures.** A. Chemical structure of an α-methylene sesquiterpene lactone, EPD. B. Chemical structure of an α-methylene carboxylic acid, EPA.

The remaining impurities in the purified sample of EPD and EPA (Figures 1A and 1B) were identified as waxes and lipids. No other sesquiterpenoid substances of similar structure to EPD and EPA were detected.

### *In vitro *cytotoxicity tests

Cell viability of normal skin fibroblasts and of cells of the ovarian cell line JC treated with the crude plant extract for 24, 48 and 72 hours at final concentrations of 1, 10 and 100 μg/mL was as follows:

The screening test for the fibroblasts with doses of 1, 10 and 100 μg/mL measured for 1 μg/mL: after 24 hours showed cell viability of 104%; after 48 hours 97%; and after 72 hours 98%; for 10 μg/ml: after 24 hours cell viability showed 100%; after 48 hours 96%; and after 72 hours 80%; and for 100 μg/mL: after 24 hours cell viability showed 98%; after 48 hours 83%; and after 72 hours 65%. At all time points (24, 48 and 72 hours) IC_50 _was greater than 100 μg/mL.

The screening test for the JC cells with doses of 1, 10 and 100 μg/mL measured for 1 μg/mL: after 24 hours showed cell viability of 98%; after 48 hours 97%; and after 72 hours 70%; for 10 μg/mL: after 24 hours cell viability showed 85%; after 48 hours 84%; and after 72 hours 21%; for 100 μg/mL: after 24 hours cell viability showed 77%; after 48 hours 84%; and after 72 hours 8%. At the time points 24 and 48 hours IC_50 _was greater than 100 μg/mL and at 72 hours IC_50 _was 2.5 μg/mL (95% confidence interval (C.I.) 0.22 to 28 μg/mL).

A similar type of biological assay was performed with the purified compound EPD at final concentrations of 1, 5 and 10 μg/mL for 24, 48 and 72 hours (Table 1). Percent of cell reduction for normal fibroblasts at 72 hours at the highest dose (10 μg/mL) was approximately 30%, while IC _50 _was greater than 10 μg/mL. Screening tests for OVCAR_3 _and SKOV_3 _cells showed that more than 50% and 80% of cells were killed at doses of 5 and 10 μg/mL, respectively.

**Table 1:** Cell viability with EPD treatment of normal fibroblasts, OVCAR_3 _and SKOV_3 _cancer cells (average (AV) and standard deviation (SD))

	% cell viability: average and standard deviation					
EPD Conc	24 hours		48 hours		72 hours	
μg/mL	AV	SD	AV	SD	AV	SD
	**Normal fibroblasts**					
1	102	2.5	107	3.9	105	3.3
5	105	6.3	108	1.6	72	2.1
10	101	10.1	112	1.8	47	4.6
	**OVCAR_3_**					
1	96	5.1	101	7.4	109	29.2
5	87	6.7	67	4.5	50	14.4
10	70	7.4	23	0.9	21	6.4
	**SKOV_3_**					
1	103	5.0	123	8.2	119	6.0
5	102	4.0	96	18.2	69	16.5
10	86	11.6	31	36.0	23	1.8

IC_50 _for OVCAR_3 _at 24 hours was 13 μg/mL (95% C.I. 10 to 18 μg/mL), at 48 hours 6.4 μg/mL (95% C.I. 5.3 to 7.8 μg/mL) and at 72 hours 5.3 μg/mL (95% C.I. 4.3 to 6.5 μg/mL).

IC_50 _for SKOV_3 _at 24 hours was 16 μg/mL (95% C.I. 9.4 to 27 μg/mL), at 48 hours 8.4 μg/mL (95% C.I. 6.7 to 11 μg/mL) and at 72 hours 6.5 μg/mL (95% C.I. 5.2 to 8.3 μg/mL).

### *In vivo *pilot experiment

Control mice only injected with the OVCAR_3 _cells, were killed when the ascites became a burden. EPD (at final concentration of 20 mg/kg b.w.) was administered i.p. twice/week for six weeks and Cisplatin (at final concentration of 5 mg/kg b.w.) was administered i.p. during 4 weeks, once/week. In general a similar cytotoxic effect was observed between EPD and Cisplatin on the OVCAR_3 _cells. However, mice treated with EPD could be kept for a much longer period of time than those mice treated with Cisplatin, for the latter the mice had lost weight significantly and had to be sacrificed after the fourth week. Moreover, following EPD treatment for 6 weeks, three mice were kept alive for another month to see if the reduced abdomen would stay of normal size. Two mice kept their normal size abdomen, whereas, after 6 weeks the abdomen of the third mouse started to increase in size (Table 2).

**Table 2:** Average abdomen size and standard deviation of BALB/c nude mice

	Average abdomen size and standard deviation (cm)					
	Control		cisplatin		EPD	
Days	AV	SD	AV	SD	AV	SD
1	2.1	0.173	2.567	0.115	2.333	0.115
7					2.4	0.173
8	2.333	0.153	2.525	0.33		
12					2.367	0.231
14			2.5	0.258		
16	2.767	0.153				
19			2.475	0.222	2.267	0.058
21	3	0.346	2.5	0.183		
26	3.1	0.141	2.1	0.1	1.967	0.208
33					2	0
36					2.267	0.058
61					2.467	0.289
63					2.533	0.321
68					2.7	0.794

**Figure 2:**
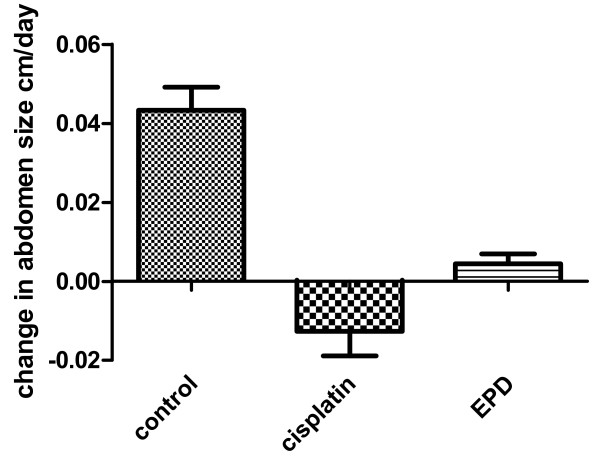
**Changes in abdomen size for control and treated mice**.

The rate of change in abdomen size for the mice was determined by linear regression (Figure 2) and statistically evaluated for significance by the unpaired t test. Control versus Cisplatin treated mice were significantly different, P = 0.023, as were control versus EPD treated mice, P = 0.025, whereas, EPD versus Cisplatin treated mice were not significantly different, P = 0.13.

## Discussion

The chemical constituents composition of aerial parts of *C. amaranthoides *have been examined once before by Zdero et al. [16]. None of the constituents reported by them were identified in the *C. amaranthoides *described in this study. The three constituents reported [16] are isomeric with the two major constituents reported in this study, EDP and EPA. The different constituents reported previously may be due to incomplete isolation and analyses or possibly the result of variation in constituent profiles of plant phenotypes. Another possible explanation is degradation on storage. Our studies have shown that freshly dried plant material is necessary as dried plant material stored for over three years was found to yield less than one-tenth of the normal yield of EDP and EPA.

For the first time the anti-cancer activity of *C. amaranthoides *has been examined. Two major sesquiterpenes with the eremophilanolide structure sub-type were identified by ^1^H-NMR and ^13^C-NMR and by mass spectrometry and by comparison with published ^1^H-NMR partial spectra as eremophila-1(10)-11(13)-dien-12,8β-olide (EPD or Xanthanodien) and eremophila-1(10),11(13)-dien-12-oic acid (EPA) [14,15]. Belonging to the family of Asteraceae, this family has contributed a large number of natural products including SL's. The alpha-methylene gamma-lactone ring is responsible for their bioactivity. Various SL's have demonstrated their anti-cancer capability in *in vitro *cell culture and by prevention of metastasis in *in vivo *animal models [6]. Thus, it is not surprising that *C. amaranthoides *extract can kill cancer cells, given the fact that one of the two isolated sesquiterpenes, EPD, shows high toxicity.

In 1972 a diastereoisomer of EPD, (3aβ,4aα,5α,9αβ)-3a,4,4a,5,6,7,9,9a octahydro4a,5-dimethyl-3-methylenenaphtho[2,3-*b*]furan-2(3H)-2-one, has been described as "naphthofuranone" by the National Cancer Institute (NCI) in their "*in vivo*" anti-tumor screening data, testing the drug against P388 Leukemia in CD2F1 mice, however, no final conclusive results were reported [17]. An allergenic sesquiterpene lactone, Alantolactone, found in "Elfdock" *Inula helenium *has been shown to be toxic to leukocytes. Although with the same molecular weight and molecular formula as EPD it belongs to the eudesmanolide structure sub-type [18]. This SL has a different chemical structure from EPD, with different positions of one methyl and one double bond.

In the present study, EPA, the other sesquiterpene isolated and identified, did not show cytotoxic effects on the ovarian cancer at concentrations up to 10 μg/mL of purified compound.

Besides the cytotoxic effects of the crude extract of *C. amaranthoides *with clear effects at 10 μg/mL (cell reduction >80%), the isolated biologically active compound EPD has been shown to have high cytotoxicity (>50%) for ovarian cancer cells at lower concentrations of 5 μg/mL (72 hours) and increased (> 60%) with a dose of 10 μg/mL (at 48 hours; Table 1). Interestingly, both the crude plant extract and EPD did show only a slight cytotoxic effect (20%-30%) on normal fibroblasts *in vitro *at a concentration of 10 μg/mL (at 72 hours). The *in vivo *pilot experiment with BALB/c nude mice (Table 2, Figure 2) did show that both EPD and Cisplatin reduced the size of the abdomen. The difference, however, was that mice treated with Cisplatin were in poor condition and became wasted compared with the EPD treated mice.

Ovarian cancer has a poor prognosis. With more than 60% of the patients presenting the disease in stage III or IV, combination chemotherapy with Platinum and Taxol after cytoreductive surgery gives the most tolerated standard regimen [19,20].

In spite of the introduction of new drugs into the management of ovarian cancer there is still need for more novel treatments.

## Conclusion

The compound EPD has shown unique cytotoxicity effects on both *in vitro *(ovarian cancer cell lines) as well as *in vivo *(mice). Interestingly, it had low cytotoxic effects on normal cells.

More studies *in vivo *are required to verify the mechanisms and mode of action of EPD, and to further validate the potential of EPD as an anti-cancer drug in ovarian cancer and other types of cancer.

## Competing interests

The authors declare that they have no competing interests.

## Authors' contributions

Data were extracted by CvH and CCD and analyzed by FD and NPMS. CCD and AWW contributed substantially to data acquisition and analysis. The paper was written by CvH and critically revised by FD and approved by all other authors including BJMZT. Revision of the manuscript was largely performed by CvH and CCD. All authors have read and approved the final manuscript.
